# Gaps in expectations and current practices of pharmacy services among doctors and patients – an exploratory study in a Sri Lankan tertiary care hospital providing free healthcare

**DOI:** 10.1186/s12913-022-08534-w

**Published:** 2022-09-12

**Authors:** J. A. L. Anjalee, V. Rutter, N. R. Samaranayake

**Affiliations:** 1 Colombo South Teaching Hospital, Kalubowila, Sri Lanka; 2 Faculty of Graduate Studies, University of Sri Jayewardenepura, Gangodawila, Nugegoda, Sri Lanka; 3 Commonwealth Pharmacists Association, London, UK; 4 Department of Pharmacy and Pharmaceutical Sciences, Faculty of Allied Health Sciences, University of Sri Jayewardenepura, Gangodawila, Nugegoda, Sri Lanka

**Keywords:** Pharmacist, Pharmacy Service, Doctors, Patients, Satisfaction, Expectation, Sri Lanka

## Abstract

The pharmacist is an important link between doctor and patient. To optimise patient care, it is essential that expectations of doctors and patients regarding pharmacy services are met. Hence the objective of this study was to assess the satisfaction levels of doctors and patients on pharmacy services currently provided, and their expectations from pharmacy services. This cross sectional study was conducted in selected clinics of a university based teaching hospital. Questionnaires developed in-house by referring previously published resources, content validated by a group of experts, and face validated through a pilot study were used. Doctors and patients of chronic disease clinics were selected for the study. All doctors involved in prescribing for more than six months, and patients or their regular care givers attending clinics for more than one year were included. Mentally incapacitated patients were excluded. An interviewee administered questionnaire was distributed to doctors and an interviewer administered questionnaire was used for patients. Exploratory factor analysis (EFA) (principal component analysis with Varimax rotation) was conducted to divide variables of the questionnaires into reliable components. Response rate of doctors was 82.3%. Among them 59.6% (50/84) doctors said that they have a good relationship with pharmacists, and 89.3% (75/84) expected communication with pharmacists more often. EFA for doctors’ perceptions resulted in four components. A statistically significant difference was observed between doctors’ expectation (95.9% - 81/84) and current practice (22.6% - 19/84) on communicating medication issues (*p*<0.001). A total of 380 patients participated. EFA for patients’ perceptions resulted in ten components. The majority considered pharmacists as an integral part of the healthcare system (98.7% - 375/380) and experts in medication (84.7% - 322/380). They further perceived that dispensed medications are safe (82.9% - 315/380) and of good quality (76.3% - 290/380). Further 95.5% (363/380) were satisfied with dispensing label information. A statistically significant difference was found between the expectations (93% - 353/380) and satisfaction levels (86.5% - 329/380) of patients on pharmacy services (*p*=0.003). According to findings, both doctors and patients held a positive perception on pharmacy services and pharmacists, but the statistically significant gap reported between expectations and current level of pharmacy service, highlighting both the potential and scope for service improvement.

## Introduction

Communication or exchanging information is an important entity in every aspect of social liaisons. Proper health communication is crucial since health information is exchanged. Health communication happens among healthcare professionals as well as between healthcare professionals and patients. The United States Department of Health and Human Services perceives that health communication is vital in each aspect of healthcare such as disease prevention, health promotion and quality of life [1].

Pharmacists play a vital role in the therapeutic management of patients. Pharmaceutical care is a crucial element in total patient care to improve the quality of life of patients. The American Pharmaceutical Association describes the pharmacy profession as “the profession responsible for the appropriate use of medications, devices, and services to achieve optimal therapeutic outcomes” [2]. Therefore the pharmacist must be responsible and concerned with providing an excellent pharmacy service to patients in order to achieve the best therapeutic outcomes for patients and enhance patient safety.

Effective professional communication among and between doctors and pharmacists is important to ensure patient safety and optimal patient care. The traditional relationship between doctors and pharmacists as prescribers and dispensers is no longer sufficient to ensure patient safety and optimum patient care [3, 4]. With the shift from “product-oriented” to “patient-oriented” services [4], the concept of pharmaceutical care evolved, requiring pharmacists to work more closely with other healthcare professionals to ensure rational use of medications [5]. Studies from Iraq and United States reveal that there is a communication gap between doctors and pharmacists, with possible reasons identified as, “lack of confidence in pharmacists, locational distance, and telephone delays” [4, 6]. Studies from Sudan and Pakistan also demonstrate that the majority of doctors (>50%) never or rarely interact with pharmacists [5, 7].

Previous studies from different countries show that doctors are more comfortable with the traditional role of the pharmacist. However, they still expect pharmacists to detect prescription errors and do consider pharmacists as experts in medication information [4, 7, 8]. An Iranian research study revealed that doctors view pharmacists playing clinical roles in patient care positively [3] while emphasizing that an increasing doctors’ awareness on the “importance of inter-professional collaboration with pharmacists” is essential in establishing and improving pharmaceutical care [3]. According to past studies, doctors felt that pharmacists’ involvement in pharmaceutical management of patients is inadequate and expected more participation in patient care [5, 7, 8].

Recently healthcare scientists have started focusing on patient satisfaction and obtaining their perceptions as a measure of evaluating the efficiency of healthcare services [9]. End user satisfaction is considered more accurate than any other standards to assess a service [9] and thus an essential part in assessing the quality of healthcare as well [10] .

When providing pharmaceutical care, pharmacists work closely with patients where the patient–pharmacist relationship is based on “caring, trust, communication, cooperation, and mutual decision making” [10]. The impact that a pharmacist can make on a patient depends on patients’ expectations, whether they trust the pharmacy services provided, and their willingness to seek pharmaceutical advice from pharmacists [10]. Successful implementation of pharmaceutical care depends on patients’ perception, satisfaction, and the professional role played by the pharmacist [11]. Many studies have been conducted to assess the public perception and satisfaction of community pharmacy services [10–16]. Some studies from Kuwait and United Arab Emirates disclose that patients perceive community pharmacists as healthcare providers not solely medication vendors or business people with monetary targets [11, 16]. In contrast, a study conducted in Poland reported pharmacists as “persons qualified to sell medications”, but not advise on healthcare [17]. According to Awad [11], in countries such as Canada, USA, Europe and Bosnia, there is a positive public perception of community pharmacy services [14]. A study in Japan revealed that patients positively perceived provision of medication information by community pharmacists [12]. Studies conducted in the middle eastern countries have reported moderate levels of satisfaction of community pharmacy services and authors suggested further development of community pharmacy services incorporating more professionalism in order to meet public expectations [10, 11, 13, 15, 16]. A study conducted in an out-patient pharmacy of a tertiary care health facility in Nigeria revealed an average satisfaction level among patients, as they expected a better quality service and appropriate counseling [18]. Also, it is suggested that pharmacists should be more knowledgeable in order to deliver better pharmaceutical care [13]. The educated personnel showed lesser satisfaction and trust in pharmacy services [18, 19].

Literature reveals that patient perception studies were mostly focused on community pharmacies and not on hospital out-patient pharmacy services. Among the few studies conducted, studies related to assess patients’ and doctors’ perceptions on the hospital pharmacy service providing free healthcare were scarce. Also past studies had not broadly divided studied variables in order to understand the major capacities of pharmacists’ relationship with doctors and patients. Hence the present study was designed to address the existing gap of identifying major capacities of pharmacists’ relationship with doctors and patients through factor analysis.

The present study was conducted as a part of a study to improve the safety of the dispensing process of the study hospital. Since pharmacists are the link between doctors and patients in medication management, knowing perceptions of doctors and patients on pharmacy services is important to enhance and optimise the process. Therefore this study aimed to assess the perceptions of doctors and patients on current pharmacy practices, and their expectations for the same, in order to identify and prioritise on the gaps, and to identify broad areas of pharmacist-doctor, and pharmacist-patient relationship through factor analysis.

## Methods

### Study design and study setting

The present cross-sectional study was carried out in a university based tertiary care hospital with a bed strength of 1110 in Colombo, Sri Lanka. The hospital serves around 2000 outpatients, 2000 clinic patients and 500 new inpatient admissions per day. The study was conducted in outpatient clinics in which patients receive long term treatment for chronic diseases. The selected clinic specialties were: Medical, Cardiology, Neurology, Rheumatology, Diabetic and Endocrinology, Paediatric, Psychiatric, Nephrology, VP - OPD (Visiting Physician – OPD), and Haematology.

### Data collection tool

An interviewee administered questionnaire was used for doctors, and an interviewer administered questionnaire was used for patients. Reliable and published sources were used to develop both questionnaires. The questionnaire to assess doctors’ perceptions was adopted from a questionnaire published in Pakistan with permission from authors [7]. The questionnaire assessed on areas of communication of medication issues between doctors and pharmacists, pharmacists’ role in providing pharmaceutical information to doctors and patients, and direct involvement of pharmacists in patient care.

The questionnaire to assess patients’ perceptions was developed based on a study in Oman with the permission from authors [10]. The questionnaire assessed patient perception on the role of pharmacists, patient expectations of services from pharmacists, patient satisfaction on current pharmacy service, and patient satisfaction on available facilities in the pharmacy.

All questions from the reference questionnaires were included except those that were not relevant to Sri Lanka. Some questions were re-worded and some new questions were added to further adapt to Sri Lanka. The questionnaires were content validated for appropriateness, clarity, relevance, and suitability to Sri Lanka by an expert panel consisting of three senior pharmacy academics, translated to Sinhala and Tamil languages (local languages used in Sri Lanka) (including back translated to English), and was face validated through a pilot study of 10 participants conducted in a local secondary care hospital (Base hospital). A five point Likert’s scale was used for responding and for scoring. A score of ‘1’ was given for the response, “Strongly Agree (SA)”, ascending to a score of ‘5’ for “Strongly Disagree (SD)”.

### Study population and sample selection

All doctors involved in prescribing to clinic patients for more than six months in the aforesaid clinics were included. According the nurse-in-charge of clinics, senior house officers deliver the major part of services at clinics with the assistance of relief house officers and house officers. Registrars, senior registrars, and consultants also participate when required (to prescribe for new patients, to see patients referred by senior house officers). Doctors not involved in prescribing to clinic patients were excluded.

Questionnaires were collected after one week followed by three personal reminders weekly before considering them as non-respondents.

According to statistics, approximately 116,522 patients attend the selected clinics during a quarter of the year. Assuming that approximately equal numbers of patients attended clinics in each month, the Raosoft online calculator (http://www.raosoft.com/ samplesize.html) was used to calculate the sample size of patients (5% margin of error, and 95% confidence level). Patients or their regular care givers who have attended the given clinic for more than one year were included. Mentally in-capacitated patients were excluded.

The questionnaire was administered to patients by the researcher while waiting in the pharmacy queue to obtain medications with the help of one Sinhala, and one Tamil speaking researcher. Every fifth patient in the queue fulfilling inclusion criteria were approached if they consented.

### Dimension reduction

Exploratory factor analysis was conducted to divide variables of the questionnaires into smaller number of components. Principal component analysis with Varimax rotation was used in dimension reduction [20]. Components with Eigen value greater than one (>1) were selected as suitable components for analysis [20]. Kaiser-Meyer-Olkin (KMO) value of sampling adequacy and Bartlett's Test of sphericity were considered to decide the suitability of data for factor analysis. The suitable KMO value for a factor analysis is 0.5 and Bartlett's Test of sphericity should be significant (*p*<0.05) [20]. Factor loadings with a value of 0.5 or more were considered when including a variable in to a particular component. A component was considered to be meaningful if a minimum of two or three variables loaded in to the given component [20]. Each component was named considering the variables loaded in each component.

### Data analysis

Questionnaires were first analysed descriptively. Average agreed and disagreed percentages were calculated for each variable. “Strongly Agree” and “Agree” responses were combined to form “agree” variable, and “Strongly Disagree” and “Disagree” responses were combined to form the “disagree” variable. Strongly agreed and agreed percentage of respondents of each component derived from dimension reduction was calculated. Medcalc online statistic calculator (https://www.medcalc.org/calc/comparison_of_means.php) was used to calculate *p* values to determine statistically significant differences between percentages.

#### Ethical clearance and informed consent

Ethical clearance was obtained from the Ethics Review Committees of University of Sri Jayewardenepura (Ref no. 64/17) and Colombo South Teaching Hospital (CSTH) (Application no. 621). Informed written consent was obtained from participants.

## Results

The response rate of doctors was 82.3%, where 84 doctors responded. A total of 380 patients participated in the study. Table 1 illustrates the demographic details of respondents.

**Table 1:** Demographic details of responded doctors and patients

Demographic details of doctors		
Demographic factor	Number of Participants (*N*=84)	
	Frequency	Percentage (%)
Gender		
Men	28	33.3
Women	56	66.7
Duration of service as a prescribing physician for clinic patients		
Less than one year	9	10.7
One to five years	21	25.0
Five to ten years	18	21.4
More than ten years	36	42.9
Position		
House officer	5	6.0
Senior house officer	37	44.0
Relief house officer	13	15.5
Medical officer	8	9.5
Registrar	14	16.7
Senior registrar	1	1.2
Consultant	6	7.1
Clinic		
Medical	35	41.7
Cardiology	7	8.3
Neurology	2	2.4
Rheumatology	3	3.6
Diabetic and endocrinology	5	6.0
Paediatric	11	13.1
Psychiatric	6	7.1
Nephrology	5	6.0
VP OPD	5	6.0
Haematology	5	6.0
Demographic details of patients		
Demographic factor	Number of participants (*N*=380)	
	Frequency	Percentage (%)
Duration of attending to clinic		
1 to 3 years	128	33.7
3 to 5 years	173	45.5
5 to 10 years	67	17.6
More than ten years	12	3.2
Gender		
Men	162	42.6
Women	218	57.4
Age		
Below 25	12	3.2
26 to 40	59	15.5
41 to 55	141	37.1
56 to 70	115	30.3
Above 70	53	13.9
Education level of the respondent		
No schooling	13	3.4
Below grade 5	31	8.2
Below grade 11	54	14.2
Passed ordinary level	177	46.6
Passed advanced level	92	24.2
Degree/ postgraduate	13	3.4
Interviewee patient or care giver		
Patient	308	81.1
Care giver	72	18.9

Exploratory factor analysis was applied to the assessment of both doctors’ perception and patient perception. A KMO value of 0.602 and 0.629 was obtained respectively. Bartlett’s test of sphericity was significant (*p*<0.001) for both. Factor analysis resulted in four and ten components with an eigenvalue more than one respectively for doctors’ perception analysis and patient perception analysis.

Components of doctors’ perception analysis (Table 2) were mainly on communication and role of pharmacists in patient care. Components of patients’ perception analysis (Table 3) were mainly on communication among patients and pharmacists, labeling, quality of medications and available facilities in the premises. Each variable loaded to a component with a value greater than 0.5 and minimum loading value was 0.527 for factor analysis on doctors’ perception, and the minimum loading value was 0.546 for factor analysis on patients’ perception. Table 4 illustrates the average agreed percentage and agreed number of participants for each tested variable to assess doctors’ and patients’ perception.

**Table 2:** Rotated component matrix – doctors’ perception and expectation

Labeling of components	Variable	Component			
		1	2	3	4
Component 1 – Doctors’ expectation to communicate pharmaceutical issues and about medications	I expect pharmacists to inform me about the medications which are out of stock.	.891			
	I expect pharmacists to inform me about the available dosage forms and strength of medications.	.882			
	I expect pharmacists to return/clarify prescriptions if they are unclear.	.698			
	I expect pharmacists to communicate with me more often than the current practice.	.696			
	I expect pharmacists to take responsibility to communicate medication related problems they discover.	.527			
Component 2 – Doctors’ perception on current status of communication on pharmaceutical issues and about medications	Pharmacists frequently ask me to clarify for them the objectives of medication regimen I have in mind for my patients		.825		
	Pharmacists frequently let me know if my patients have experienced some problem with their medications		.796		
	Pharmacists routinely inform me about more cost-effective alternatives to the medications I prescribe		.785		
	Pharmacists routinely inform me if they discover medication related problems with my prescriptions.		.739		
Component 3 - Doctors’ perception on pharmacists’ suitability in providing pharmaceutical information to patients and prescribers	I routinely direct my patients to pharmacist to counsel regarding the safe and appropriate use of their medications.			.767	
	In my experience, pharmacists are a reliable source of information on medications			.757	
	I frequently contact pharmacist to inquire about medication interactions and side effect related queries.			.651	
	I expect pharmacists to educate my patients about the safe and appropriate use of their medication			.602	
	Pharmacists usually speak with confidence about pharmaceutical matters.			.547	
Component 4 - Doctors’ perception on involvement of pharmacists in therapeutic management of patients	I expect pharmacists to assist my patients in selecting appropriate non-prescription medications.				.809
	Pharmacists appear willing to communicate medication related problems they discover.				.725
	I expect pharmacists to monitor my patients’ response to medications and let me know if a patient encounters any medication-related problem				.686
	I expect pharmacists to assist me in designing medication regimen plans for my patients				.631

Extraction method – Principal Component Analysis

Rotation method – Varimax with Kaiser normalization

**Table 3:** Rotated component matrix – patients’ satisfaction and expectation

Labeling of components	Variable	Component									
		1	2	3	4	5	6	7	8	9	10
Component 1 - Patients’ expectation to gain detailed information on their medication from the pharmacists	I expect pharmacists to check my prescriptions for medication related problems before dispensing the medication.	.817									
	I expect pharmacists to tell me the name and strength of each medication	.921									
	I expect pharmacists to tell me the names of each medication and what they are used for.	.897									
	I expect pharmacists to give me instructions in a language preferred by me.	.784									
Component 2 - Patients’ satisfaction on medication labeling information currently given by the pharmacists	I am satisfied with the written label information given by pharmacists on how to use medications		.925								
	I am satisfied with the readability of label instructions given by pharmacists		.941								
	I am satisfied with the understandability of label instructions given by pharmacists		.906								
Component 3 - Patients’ expectation to resolve their queries on medication with the pharmacists	I expect pharmacists to give me verbal instructions on how to take my medications in addition to written label.			.835							
	I expect pharmacists to warn me of any possible side effects and how to prevent it			.897							
	I expect pharmacists to help clarify my questions related to medications			.836							
	I expect pharmacists give me trusted information on the use of medications			.764							
Component 4 - Patients’ trust and satisfaction on the current procedure of dispensing medication by the pharmacists	I am satisfied with the questions asked by pharmacists before dispensing medications like name of the patient, any history of previous drug allergy, disease details, etc.				.849						
	I am satisfied with the privacy maintained by pharmacists while discussing and dispensing medication				.952						
	I am satisfied with the level of knowledge that pharmacists possess in medication related issues				.916						
Component 5 - Satisfaction and perception of patients on professional relationship of pharmacists with patients	I do not consider pharmacists as a mere dispenser of medications					.546					
	I am satisfied with the kind of responses pharmacists provide for questions I ask about my medications					.821					
	I am satisfied with the duration of time spend by pharmacists with each patient					.872					
	I am satisfied with the relationship that pharmacists try to maintain with the patients					.779					
Component 6 – Patient satisfaction on safety and quality of dispensed medications	I am satisfied about the safety of medications dispensed by pharmacists						.963				
	I am satisfied with the quality of the medications dispensed by pharmacists						.966				
Component 7 - Patient perception on the role of pharmacists in healthcare system	I consider pharmacists as experts in matters related to medications.							.877			
	I consider pharmacists as an integral part of the healthcare system like doctors and nurses.							.852			
Component 8 - Satisfaction of patients on the available facilities in the pharmacy	I am satisfied with the number of pharmacists and number of counters available to dispense the medications								.789		
	I am satisfied with available facilities in the pharmacy waiting area for patients								.759		
	I am satisfied about the time spent at the pharmacy for obtaining medications								.661		
Component 9 - Patients’ satisfaction on detailed information provided by the pharmacist on medications and other health issues	I am satisfied with the kind of information pharmacists provide on disease and other health issues along with information on drugs									.787	
	I am satisfied with the information pharmacists give me on name of the medication and what it is used for									.777	
Component 10 - Patients’ satisfaction on verbal information of medications given by pharmacists while dispensing	I am satisfied with the information given by pharmacists when there is a change in medications or doses in my prescription.										.668
	I am satisfied with the verbal information given by pharmacists on how to use medications										.704
	I am satisfied with the accuracy of information given by pharmacists										.609

Extraction method - Principal Component Analysis

Rotation method - Varimax with Kaiser normalization

**Table 4:** Average percentage and number of agreed participants for each tested variable

Tested variable	Agreed participants^a^	
	Frequency (Total N=84)	Percentage (%)
**Assessment of doctors’ perception and expectation**		
I frequently contact pharmacist to inquire about availability of particular medicines, dosage forms and strengths	39	46.4
I frequently contact pharmacist to inquire about medicine interactions and side effect related queries	4	4.8
I have a good relationship with pharmacists in the hospital	50	59.6
In my experience, pharmacists are a reliable source of information on medicines	42	50
I routinely direct my patients to pharmacist to counsel regarding the safe and appropriate use of their medicines.	12	14.3
Pharmacists routinely inform me if they discover medicine related problems with my prescriptions	45	53.6
Pharmacists routinely inform me about more cost-effective alternatives to the medicines I prescribe	3	3.6
Pharmacists frequently ask me to clarify for them the objectives of medicine regimen I have in mind for my patients	8	9.5
Pharmacists frequently let me know if my patients have experienced some problem with their medicines	20	23.8
Pharmacists usually speak with confidence about pharmaceutical matters	28	33.4
Pharmacists appear willing to communicate medicine related problems they discover	35	41.7
I expect pharmacists to take responsibility to communicate medicine related problems they discover	77	91.6
I expect pharmacists to return/clarify prescriptions if they are unclear	84	100
I expect pharmacists to assist me in designing medicine regimen plans for my patients	35	41.7
I expect pharmacists to educate my patients about the safe and appropriate use of their medication	78	92.9
I expect pharmacists to monitor my patients’ response to medicines and let me know if a patient encounters any medicine-related problem	39	46.4
I expect pharmacists to assist my patients in selecting appropriate non-prescription medications	40	47.6
I expect pharmacists to inform me about the available dosage forms and strength of medicines	83	98.9
I expect pharmacists to inform me about the medicines which are out of stock	84	100
I expect pharmacists to communicate with me more often than the current practice	75	89.3
**Assessment of patients’ satisfaction and expectation**		
	(Total *N*=380)	
I consider pharmacists as experts in matters related to medicines	322	84.7
I do not consider pharmacists as a mere dispenser of medicines	320	84.2
I consider pharmacists as an integral part of the healthcare system like doctors and nurses	375	98.7
I expect pharmacists to check my prescriptions for medication related problems before dispensing the medication	344	90.5
I expect pharmacists to tell me the name and strength of each medicine	354	93.2
I expect pharmacists to tell me the names of each medicine and what they are used for	355	93.4
I expect pharmacists to give me instructions in a language preferred by me	313	82.4
I expect pharmacists to give me verbal instructions on how to take my medicines in addition to written label	371	97.6
I expect pharmacists to warn me of any possible side effects and how to prevent it	365	96
I expect pharmacists to help clarify my questions related to medicines	361	95
I expect pharmacists give me trusted information on the use of medicines	366	96.3
I am satisfied with the questions asked by pharmacists before dispensing medications like name of the patient, any history of previous drug allergy, disease details, etc.	295	77.6
I am satisfied with the privacy maintained by pharmacists while discussing and dispensing medication	275	72.4
I am satisfied with the level of knowledge that pharmacists possess in medicine related issues	332	87.4
I am satisfied with the kind of responses pharmacists provide for questions I ask about my medicines	334	87.9
I am satisfied with the duration of time spend by pharmacists with each patient	295	77.6
I am satisfied with the relationship that pharmacists try to maintain with the patients	299	78.7
I am satisfied with the kind of information pharmacists provide on disease and other health issues along with information on drugs	337	88.7
I am satisfied with the information pharmacists give me on name of the medicine and what it is used for	338	89
I am satisfied with the information given by pharmacists when there is a change in medicines or doses in my prescription	304	80
I am satisfied with the verbal information given by pharmacists on how to use medicines	344	90.6
I am satisfied with the written label information given by pharmacists on how to use medicines	363	95.6
I am satisfied with the readability of label instructions given by pharmacists	364	95.8
I am satisfied with the understandability of label instructions given by pharmacists	362	95.2
I am satisfied with the respect and courtesy that pharmacists extended to me	206	54.2
I am satisfied with the number of pharmacists and number of counters available to dispense the medicines	19	5
I am satisfied with available facilities in the pharmacy waiting area for patients	53	14
I prefer if a numbering system was available to serve patients at the pharmacy than the ‘next in line’ practice	361	95
I am satisfied with the accuracy of information given by pharmacists	343	90.2
I am satisfied about the safety of medicines dispensed by pharmacists	315	82.9
I am satisfied with the quality of the medicines dispensed by pharmacists	290	76.3
I am satisfied about the time spent at the pharmacy for obtaining medicines	6	1.6
I am satisfied with the overall service provided by this pharmacy	293	77.1

^a^Strongly agree+Agree

Among the participants, 59.6% (50/84) doctors said that they have a good relationship with pharmacists in the hospital, and 89.3% (75/84) expected pharmacists to communicate more often than currently practiced. Only 14.3% (12/84) doctors agreed that they routinely direct their patients to a pharmacist to counsel on safe and appropriate use of medications. All the doctors (100%) expected unclear prescriptions to be returned back to them, and expected pharmacists to inform of out of stock medications. Only 41.7% (35/84) expected pharmacists to assist doctors in designing medication regimen plans to patients.

Among patient respondents, 98.7% (375/380) considered pharmacists as an integral part of the healthcare system, and 84.7% (322/380) considered pharmacists as experts in medication related matters. The majority of patients believed that dispensed medications were safe (82.9% - 315/380) and of good quality (76.3% - 290/380). Except for a few patients, the majority (95% - 361/380) preferred a numbering system for obtaining medications rather than the current ‘next in line’ practice. There was a 77.1% (293/380) satisfaction level among patients about the overall service provided by the pharmacy, and 54.2% (206/380) were satisfied with the respect and courtesy extended by pharmacists.

Table 5 shows the average agreed percentage of participants; i.e. who responded as ‘strongly agreed’ or ‘agreed’ to each of the components derived from factor analysis. The majority of the doctors (95.9%, 81/84) expected pharmacists to communicate about medication issues with them whereas only 22.6% (19/84) doctors agreed that pharmacists do so in current practice (p<0.001).

**Table 5:** Average agreed percentage of each tested component derived from factor analysis

Component	Average agreed percentage %^**a**^ (n)
**Assessment of perception and expectation of doctors**	
Component 1 – Doctors’ expectation to communicate pharmaceutical issues and about medications	95.9 (81)
Component 2 – Doctors’ perception on current status of communication on pharmaceutical issues and about medications	22.6 (19)
Component 3 - Doctors’ perception on pharmacists’ role in providing pharmaceutical information to patients and prescribers	39.1 (33)
Component 4 - Doctors’ perception on involvement of pharmacists in therapeutic management of patients	44.3 (37)
**Assessment of satisfaction and expectation of patients**	
Component 1 - Patients’ expectation to gain detailed information on their medication from the pharmacists	89.8 (341)
Component 2 - Patients’ satisfaction on medication labeling information currently given by the pharmacists	95.5 (363)
Component 3 - Patients’ expectation to resolve their queries on medication with the pharmacists	96.2 (366)
Component 4 - Patients’ trust and satisfaction on the current procedure of dispensing medication by the pharmacists	79.1 (301)
Component 5 - Satisfaction and perception of patients on professional relationship of pharmacists with patients	82.1 (312)
Component 6 – Patient satisfaction on safety and quality of dispensed medications	79.6 (302)
Component 7 – Patient perception on the role of pharmacists in healthcare system	91.7 (348)
Component 8 - Satisfaction of patients on the available facilities in the pharmacy	6.8 (26)
Component 9 - Patients’ satisfaction on detailed information provided by the pharmacist on medications and other health issues	88.8 (337)
Component 10 - Patients’ satisfaction on verbal information of medications given by pharmacists while dispensing	86.9 (330)

^a^Responded as either ‘Strongly agree’ or ‘Agree’

In the factor analysis of patient perception, components 1 and 3 (total agreed % = 93, 353/380) resembled expectations of patients on services offered by hospital pharmacists. Components 2, 4, 5, 9 and 10 (total agreed % = 86.5, 329/380)) resembled satisfaction of patients on services offered by hospital pharmacists. A statistically significant difference (*p*=0.003) was observed between expectation and satisfaction of patients about the services of hospital pharmacists.

## Discussion

Although 59.6% (50/84) of doctor respondents from the present study stated that they have a good relationship with pharmacists, 46.4% (39/84) said that they frequently contacted pharmacists to enquire about the availability of particular medications, dosage forms, and strengths. However, the majority of doctors expected pharmacists to contact them more frequently than currently practiced, and to communicate medication-related and prescription-related issues. A great percentage of patients considered pharmacists as experts in medications (84.7% - 322/380)) and as an integral part of the healthcare system (98.7% - 375/380). Although there was a statistically significant difference (*p*=0.003) between the expectations and satisfaction, a large percentage (86.5%, 329/380) of patients were satisfied with the present service of pharmacists. There was a 77.1% (293/380) overall satisfaction among patient respondents.

Although pharmacists play a significant role in patient care, the concept of pharmaceutical care is at a primary stage in most developing countries [4, 7], including Sri Lanka. Most studies demonstrated the importance of doctor-pharmacist relationship in developing pharmaceutical care and to implement clinical pharmacy services where pharmacists could provide ‘direct patient care’ [4, 7, 8]. Nearly half of the responding doctors in the present study stated that they have a good relationship and frequent contact with pharmacists. However, the majority expected pharmacists to contact them more frequently than was currently practiced, and to communicate medication-related and prescription-related issues with them. The results from other studies reflect the findings of this study and also reported that doctors interacted more with pharmacists to find out about the availability of particular medications [4, 7]. Interestingly perhaps, one study [21] stated that doctors did not have a clear knowledge regarding what to expect from pharmacists. Younger doctors who graduated recently (less than 10 years ago) had more expectations of pharmacists [21].

Avoiding prescription errors from reaching patients was another service expected of pharmacists by doctors [3–7]. Since the pharmacist is the last healthcare professional in the medication use process in ambulatory care, it is an essential responsibility of pharmacists to act as a safety net and help avoid medication errors. A statistically significant gap (p<0.001) between the doctors’ expectation and present practice in relation to communication should encourage Sri Lankan hospital pharmacists to enhance professional communication with doctors, which will in turn help to develop the pharmacy services in the country.

Comprehensive patient education is important in improving patient compliance and thereby optimising therapeutic outcomes of medication therapy. Patients from the present study also expected pharmacists to give detailed information on medications (89.8%, 341/380) and resolve their queries (96.2%, 366/380). Ranelli et al., [6] stated that doctors consider themselves to be responsible for patient education. However, pharmacists also have an important role on more medication oriented aspects of patient education, allowing them to build on and reinforce messages received during the very limited time a patient is able to spend with the doctor. In many countries pharmacists do counselling patients frequently but there is a disparity in practice across countries. This was also revealed by a study conducted in Pakistan [7]. It is clear that a better collaboration and understanding is needed between doctors and pharmacist in establishing robust patient counseling programmes.

According to past studies, the doctors were positive in varying percentages towards pharmacists being involved in patient-centered clinical services such as suggesting non-prescription medication (32%, 83.5%), therapeutic regime monitoring (33%, 59.8%), and patient counseling (34.7%, 65.4%) [3, 5–7, 21]. Good inter-professional relationships with doctors is significant in establishing direct patient care roles such as in clinical pharmacy, especially in countries where the concept of clinical pharmacy is not really practiced. A study in Iraq revealed that the majority of doctors were comfortable with pharmacists treating minor illness and suggesting prescription medications to physicians (75% and 67% respectively) [4]. According to a study from Pakistan, 76% doctors considered pharmacists as knowledgeable drug therapy experts [7]. However, a study in Kuwait stated that doctors are less comfortable with pharmacists providing direct patient care [8]. Results of the present study showed mixed perceptions where, on average 45.2% of doctors expected pharmacists to be involved in direct patient care roles such as: assisting doctors to design medication regimen plans, monitoring patients’ response to medications and assisting patients to select appropriate non-prescription medications. Hence ongoing effective professional communication and wider exposure to roles of each other may help pharmacists in developing countries to establish professional pharmaceutical care services supported by the doctors.

It is said that “perception and psychological acceptance of the medications provided” is considered as a significant and prioritised component of effective medication treatment [15]. Assessing patient perception and satisfaction is identified as a most reliable indicator of quality of a healthcare service [9].

The majority of respondents in this study considered pharmacists as experts in medications (84.7%, 322/380) and not mere vendors of medications, in contrast to two other studies conducted in Poland and UAE where community pharmacists were considered as mere retailers of medications [16, 17]. However, other studies conducted in community pharmacies globally revealed that pharmacists are trusted professionals and considered as healthcare professionals [10, 11, 14, 19]. This is evidence that the public in many parts of the world perceive pharmacists as a significant part of healthcare, whether in hospital or community pharmacy. This leaves a great responsibility to pharmacists all over the world to offer quality pharmaceutical care for patients and promote the professionalism of pharmacy service.

Proper labeling of medications is important to help ensure patients take medicines correctly and as intended. It is said that only about 50% of medications are taken correctly by patients even in countries that have a well-established labeling systems [22]. The present study shows 95.6% patient satisfaction on label information given by pharmacists as in other studies that also demonstrated a fair percentage of patient satisfaction (72% - 96%) in relation to labeling by the pharmacy [11, 16, 18].

The present study reported 77.1% of overall satisfaction of patients in the pharmacy service in comparison to other studies that reported various satisfaction levels categorised as good [14, 18], poor [11, 17] or moderate [10, 19]. However, the majority of patients were not satisfied with the available facilities (satisfaction - 14%, 53/380) in patient waiting area and numbers of counters and pharmacists (satisfaction- 5% - 19/380). Although the study setting was a university based teaching hospital in Sri Lanka where health services are provided at no cost, the dissatisfaction on facilities must be explored and improved. Also the reported statistically significant disparity between expectation and satisfaction of patients (*p*=0.003) reveals a clear gap. Interventions are needed to improve communication gaps with patients.

Conducting the study with doctors and patients of a selected number of clinics in only one study setting is a limitation of this study.

## Conclusion

Pharmacists are an important bridge between doctors and patients. Hence knowing the perceptions and expectations of both is an important factor in enabling improved pharmaceutical services.

The present study highlights that pharmacists are considered as a vital part of patient care both by doctors and patients, and that the opportunity and need exists for pharmacists to upgrade the pharmacy service and enhance the role of pharmacists as trusted healthcare professionals. It also highlights the need for better understanding and communication between the professions. Opportunities for interprofessional learning, beginning in the undergraduate years would be of benefit to enhancing understanding and communication, building a strong foundation for a supportive professional relationship that can help develop future services to leverage the knowledge and skills of both professions for the benefit of patients.

Dimension reduction (factor analysis) derived broad aspects (different components) of doctor – pharmacist, and patient – pharmacist relationships that can be used in future assessment of these relationships. Conducting periodic surveys to assess patients’ and doctors’ satisfaction and expectations of pharmacy services in healthcare institutions is important in countries such as Sri Lanka where pharmacy service is in nascent stage. The validated tools used in this study and components derived through factor analysis may be helpful for this purpose in Sri Lanka and other countries with pharmacy services at a similar stage of development.

## Data Availability

All data generated or analysed during this study are included in this article
